# Curcumin Reduces Colorectal Cancer Cell Proliferation and Migration and Slows In Vivo Growth of Liver Metastases in Rats

**DOI:** 10.3390/biomedicines9091183

**Published:** 2021-09-08

**Authors:** Borja Herrero de la Parte, Mikel Rodeño-Casado, Sira Iturrizaga Correcher, Carmen Mar Medina, Ignacio García-Alonso

**Affiliations:** 1 Department of Surgery and Radiology and Physical Medicine, University of The Basque Country, ES48940 Leioa, Spain; mrodeno001@ikasle.ehu.eus (M.R.-C.); ignacio.galonso@ehu.eus (I.G.-A.); 2 Biocruces Bizkaia Health Research Institute, ES48903 Barakaldo, Spain; 3 Department of Clinical Analyses, Osakidetza Basque Health Service, Galdakao-Usansolo Hospital, ES48960 Galdakao, Spain; sira.iturrizagacorrecher@osakidetza.eus (S.I.C.); mariadelcarmen.marmedina@osakidetza.eus (C.M.M.)

**Keywords:** curcumin, in vitro proliferation, wound healing test, partial hepatectomy, liver metastases

## Abstract

Background: New therapeutic approaches are an essential need for patients suffering from colorectal cancer liver metastases. Curcumin, a well-known plant-derived polyphenol, has been shown to play a role in the modulation of multiple signaling pathways involved in the development and progression of certain cancer cells in vitro. This study aims to assess the anti-tumor effect of curcumin on CC531 colorectal cancer cells, both in vitro and in vivo. Methods: On CC531 cultures, the cell viability and cell migration capacity were analyzed (wound healing test) 24, 48, and 72 h after treatment with curcumin (15, 20, 25, or 30 µM). Additionally, in WAG/RijHsd tumor-bearing rats, the total and individual liver lobe tumor volume was quantified in untreated and curcumin-treated animals (200 mg/kg/day, oral). Furthermore, serum enzyme measurements (GOT, GPT, glucose, bilirubin, etc.) were carried out to assess the possible effects on the liver function. Results: In vitro studies showed curcumin’s greatest effects 48h after application, when all of the tested doses reduced cell proliferation by more than 30%. At 72 h, the highest doses of curcumin (25 and 30 µM) reduced cell viability to less than 50%. The wound healing test also showed that curcumin inhibits migration capacity. In vivo, curcumin slowed down the tumor volume of liver implants by 5.6-fold (7.98 ± 1.45 vs. 1.41 ± 1.33; *p* > 0.0001). Conclusions: Curcumin has shown an anti-tumor effect against liver implants from colorectal cancer, both in vitro and in vivo, in this experimental model.

## 1. Introduction

Over the last years, great efforts have been devoted to identifying new compounds with anticancer properties. An important source for these potential compounds lies in botany. Several plant-derived compounds with anti-cancer properties have been identified, such as resveratrol, lycopene, silibinin, curcumin, artemisinin, berberine, camptothecin, and other plant-derived lipids [1,2].

Curcumin is a well-known plant-derived polyphenol with a wide range of activities, such as antibacterial, anti-inflammatory, and hepatoprotective properties. In vitro studies with several cancer cell lines have proven curcumin to inhibit proliferation, such as on MCF-7 human breast tumor cells [3]. It has also been observed that curcumin can improve the efficacy of other therapies, such as Paclitaxel on rat glioma C6 cells [4], and of 5-FU and/or oxaliplatin in BGC-823 human gastric cancer cell line [5] or in HCT116 human colon cancer cells [6]. In this cell line, it has been found that curcumin inhibits the cell cycle, activates p53 (only in p53+/+ cells) and p21 [7], and triggers cellular senescence (irreversible growth arrest of proliferating cells) through the activation of the lysosomal enzyme senescence-associated-β-galactosidase (SA-β-gal) and the upregulation of the p21 protein [7,8]. Other studies have also shown that curcumin-induced apoptosis is related to oxidative stress caused by the production of superoxide anion, which contributes to p53-independent cellular cytotoxicity [9]. These findings were further corroborated in other cell lines (COLO-205), showing that curcumin induces cytotoxicity and apoptosis in a dose-dependent manner. In addition, curcumin promotes the production of reactive oxygen species and Ca2+, and induces the caspase-3 activity [10]. In in vivo animal studies using cancer cells implants (most of them in animals lacking immune system), curcumin reduces the growth of the implants and the appearance of metastases [11,12,13,14,15]. It has also been demonstrated that curcumin reduces tumor invasion and metastatic growth by, for example, reducing the activity of matrix metalloproteinases (MMPs) 2 and 9, or blocking nuclear translocation of nuclear factor kappa B (NFκB) by inhibiting the inhibitor of kappaB kinase [11,12,13]. This molecular pathway is also involved in the prevention of liver damage after exposure to radiotherapy [16] by regulating oxidative stress damage and reducing the accumulation of reactive oxygen species, which are well known to contribute to tumor progression by promoting cell transformation, proliferation, and the survival of tumor cells [17].

Despite these promising results on the use of curcumin in different cell lines, there is a lack of evidence on the use of curcumin in metastatic colorectal carcinoma cell lines. Colorectal cancer (CRC) is the third most common cancer worldwide in both sexes, and the second most common cancer in terms of mortality [18]. Moreover, the global incidence of colorectal cancer is projected to increase to 2–5 million new cases by 2035 [19]. The liver is the major site of distal spread of tumor disease, and approximately 15–25% of CRC patients will have metastases at the time of primary tumor diagnosis [20].

The multidisciplinary approach to colorectal liver metastases (CRCLM), as well as advances in systemic chemotherapy, have increased the overall survival. The current trend in the systemic treatment of CRCLM is to combine systemic chemotherapy (5-fluorouracil [5-FU], irinotecan, or oxaliplatin, alone or in combination) with targeted therapies (EGFR- or VEGF-targeting monoclonal antibodies), which allows for increased response rates and conversion of patients with initially unresectable CRCLM [21,22,23,24]. However, many of these targeted therapies are not fully effective due to the existence of subclones that bear mutations that confer resistance to the therapy. These more resistant subclones will therefore expand and cause recurrence [25].

So far, curcumin has shown promising results in several animal and in vitro models, however none of them include all the natural phases of metastases development. We have followed the “next step”, using a model of liver cancer implants originated from cell migration from the spleen to the liver, and a surgical procedure of partial liver hepatectomy to check if curcumin could be useful in a clinical setting.

## 2. Materials and Methods

All protocols were approved by the institutional review board (IRB) for animal research (CEEA) (ID: M20/2020/136) and for research with biological agents (CEIAB) (ID: M30/2020/256) of the University of the Basque Country (UPV/EHU). All animal procedures were performed in accordance with the current national and European legislation on animal experimentation.

### 2.1. Cell Preparation

CC531 colocarcinoma cells (CLS Cell Lines Service GmbH, Eppelheim, Germany) were used in all of the experiments. Cells were cultured at 37 °C and 5% CO_2_ in RMPI (Thermo Fisher Scientific Inc., Waltham, MA, USA) containing 10% fetal calf serum (Pan Biotech, Aidenbach, Bavaria, Germany), and supplemented with penicillin (100 UI/mL), streptomycin (100 μg/mL), and amphotericin B (0.25 μg/mL) (Calbiochem, Burlington, MA, USA).

### 2.2. Proliferation Assays

In 96-well culture plates (Eppendorf, Eppendorf, Hamburg, Germany), 2500 cells were seeded in each well (100 µL). After 24 h, curcumin (Acrós Organics, Geel, Antwerp, Belgium)diluted in dimethyl sulfoxide (DMSO) (0.001–0.005% v/v) (Sigma-Aldrich, St. Louis, MO, USA) was added at 15, 20, 25, or 30 µM. Cell viability was assessed with Prestoblue Cell Viability Reagent™ (Invitrogen, Carlsbad, CA, USA) 24, 48, and 72 h after curcumin treatment. Fluorescence was measured using a SynerGy HT plate fluorometer (BioTEK, Winooski, VT, USA). Proliferation was assessed in triplicate, in independent experiments, with 16 wells for each experimental condition.

### 2.3. Wound Healing Test

To detect cell migration, 50,000 cells/mL in 70 µL were seeded in each double-well silicone Culture Insert-2 Well (ibidi GmbH, Munich, Bavaria, Germany). After 24 h, the insert was removed and non-adherent cells were washed out with phosphate buffered saline. Then, fresh medium containing 0, 15, 20, 25, or 30 µM curcumin was added. Over 72 h, under a light microscope at 40× magnification, pictures of the cultures were taken at different time intervals. The wound area and five measurements of the gap between the two opposite cell fronts were measured from each picture (Leica Application Suite software, Leica Microsystems, Wetzlar, Hesse, Germany). These measurements were carried out in two independent replicates, with 12 wells for each experimental condition.

### 2.4. Tumor Induction

For tumor induction, CC531 cells were suspended at 500,000 cell/mL in Hank’s solution. Twenty rats were anaesthetized with 1.5% isoflurane, and were administered with meloxicam (2 mg/kg, sc). Through a midline laparotomy (20–25 mm), the left portal vein branch was exposed and occluded with a Yasargil microvascular clip to avoid losing tumor cells in the left lateral lobe. Then, a single injection of 0.5 mL of cell suspension (500,000 cell/mL in Hank’s solution) was administered into the spleen. Cells were allowed to migrate from the spleen for 5 min; then, the spleen was removed to avoid a “primary tumor” in the organ. Five minutes later, a 40% hepatectomy was performed, the laparotomy was closed, and buprenorphine (0.05 mg/kg, sc) was administered (Figure S1). Animals were checked daily throughout the period of tumor development (28 days), following IRB-approved humane endpoint criteria. A single in vivo experiment, without replicates, was performed following the principle of the 3Rs (replacement, reduction, and refinement).

### 2.5. Treatment Administration

Curcumin suspension in sesame oil (ranging 0.6–0.8 mL, according to animal weight) was prepared fresh every day to avoid possible degradation of the curcumin. Control-group animals were given the same volume of sesame oil (vehicle). Both the drug and vehicle were administered orally, using a 18 G polypropylene cannula (Instech, Plymouth Meeting, Pennsylvania, USA). Curcumin (200 mg/kg [26]) and vehicle were administered starting one day before cell injection (day −1) and ending on the day of sacrifice (day +28) (Figure S3).

### 2.6. Blood and Tissue Collection and Analysis

On day 28, the animals were sacrificed. Under isoflurane, a wide laparotomy was performed and blood (6–7 mL) was retrieved from the inferior vena cava. Serum was analyzed for alanine transaminase (ALT), aspartate transaminase (AST), alkaline phosphatase (ALP), total bilirubin (TBil), glucose, cholinesterase, cholesterol, total protein (TP), and albumin (all chemical reagents were purchased from Roche Diagnostics GMBH, Rotkreuz, Zug, Switzerland). To obtain the biochemical serum values of the control group, blood samples were obtained from another six non-tumor-bearing and untreated animals.

The whole liver was removed and fixed in 4% formaldehyde. After 24 h, each liver lobe was sliced into 1.5–2 mm thick sections, and photographs were taken from each slice to measure the total area occupied by the liver parenchyma or tumor tissue (ImageJ 1.8.0_112; National Institutes of Health, Bethesda, MD, USA). Then, the areas from all the slices were summed up, and multiplying that figure by the mean thickness of the slices, the volume was assessed (both for hepatic and tumor tissue).

### 2.7. Statistical Analysis

As the normality of our data was confirmed using the Kolmogorov−Smirnov test, the results were described using mean and standard deviation (SD). A two-tailed t-test was used when comparing the data from the two groups. To compare three or more groups, ANOVA (with the Newman−Keuls multiple comparison test for between-groups comparison) was used. AA 95% confidence level was accepted as significant. Analyses were performed with GraphPad Prism 6.04 (GraphPad Software, San Diego, CA, USA).

## 3. Results

### 3.1. In Vitro Experiments

**Figure 1:**
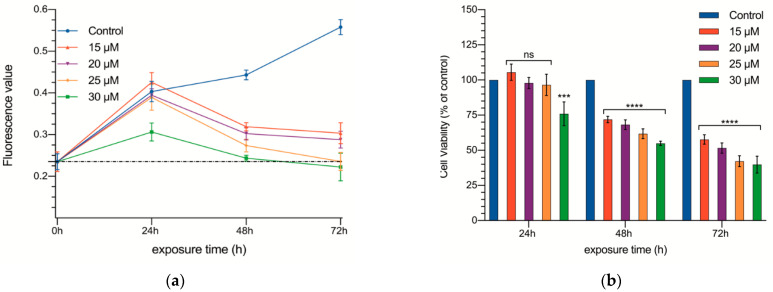
Cell proliferation of CC531 rat colorectal cancer cells as a function of time. Absolute fluorescence values (**a**) and percentage of control (**b**) and cultures supplemented with 15, 20, 25, and 30 µM of curcumin at 24, 48, and 72 h. The dotted line of the figure a shows fluorescence values prior to curcumin exposure. Results are expressed as mean and standard deviation (SD) from three replicates (16 well per replicate), and have been statistically analyzed by a one-way ANOVA test, followed by Newman−Keuls multiple comparison test for between-group comparisons. Asterisks indicate statistical significance: ***: *p* < 0.001; **** *p* < 0.0001; ns: *p* > 0.05.

In a series of experiments, DMSO proved to be of no effect on cell growth at the concentrations used to dilute curcumin (Figure S2). Once DMSO was shown to be non-toxic, treatment with different doses of curcumin (15, 20, 25, and 30 µM) was carried out (Table S1 and Figure 1a,b). Prior to treatment, the basal fluorescence values detected in the cell cultures were 0.235 ± 0.095 arbitrary units (AU).

The effect of the different concentrations of curcumin on cell proliferation are summarized in Table S1. After the first 24 h, only the highest concentration (30 µM) slowed down cell proliferation (0.403 ± 0.122 vs. 0.306 ± 0.108 AU, *p* < 0.001). Then, in the following measurements (48 and 72 h), all of the concentrations tested hindered cell proliferation, and the effect showed a dose dependent pattern (Figure 1). Not only was no proliferation observed between 24 and 48 h, but even the total number of cells in the well decreased significantly.

The cultures receiving the two smaller concentrations (15 and 20 µM) reached a plateau starting at 48 h (no further reduction in the number of cells was observed; 0.319 ± 0.040 and 0.302 ± 0.061 AU, respectively), while the other two concentrations continued decreasing the number of cells in the cultures (0.235 ± 0.08 and 0.222 ± 0.088 AU, respectively; in fact, at 72 h, the number of cells was practically identical to that at 0 h).

**Figure 2:**
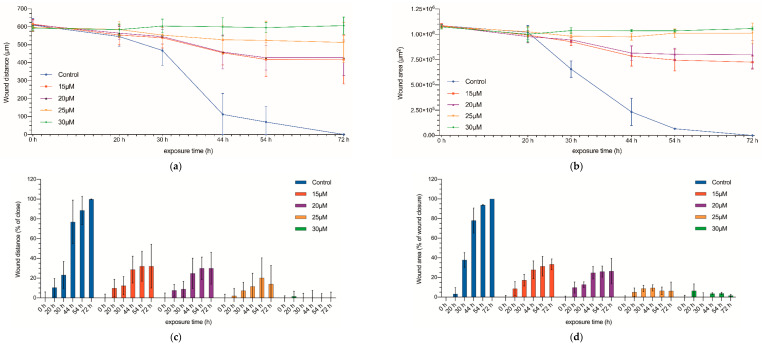
Wound healing assay. Wound distance (**a**) and wound area (**b**) measured at 0, 20, 30, 44, 54, and 72 h after being exposed to curcumin (15, 20, 25, and 30 µM). Wound-closure rate (**c**); wound-area closure rate (**d**) at 0 set to 0% and the percentage of wound closure has been calculated at 20, 30, 44, 54, and 72 h. Results are expressed as mean and standard deviation (SD) from three replicates (12 well per replicate), and have been statistically analyzed by a one-way ANOVA test, followed by Newman−Keuls multiple comparison test for between-group comparisons.

**Figure 3:**
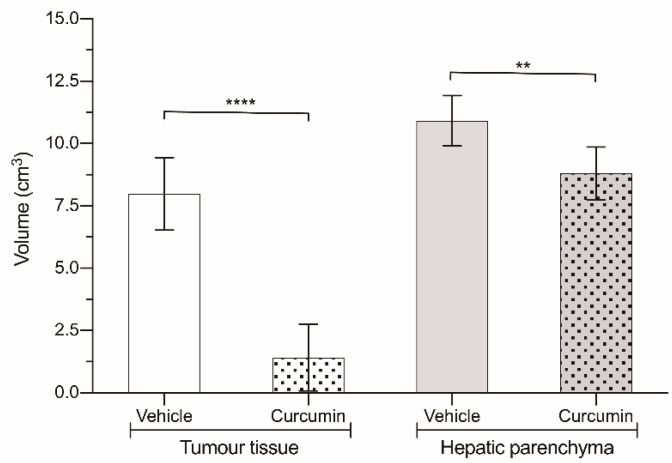
Volume of liver (grey) and tumor (white) tissue in animals treated with 200 mg/kg curcumin (dotted bars) and in animals treated with vehicle (solid bars). Results are expressed as mean and standard deviation (SD) from a single replicate (10 animals per group) and statistically analyzed by two-tailed t-test. Asterisks indicate statistical significance: **: *p* < 0.01; **** *p* < 0.0001.

Once the inhibitory effect on the proliferation of CC531 cells was proven, we used the wound healing test to assess the invasiveness capacity of the treated cells (Figure 2 and Figure S3). The initial gap between the two opposite cell fronts was 1.08 × 10^6^ µm^2^, with a mean distance of 605 ± 26.35 µm (Figure 2 and Figure 3). In no-treated cultures, after 30 h a significant decrease in the gap could be observed, and by 72 h, both fronts collided completely. On the other side, those cultures treated with the highest concentrations of curcumin (25 and 30 µM) kept the edge separation constant throughout the experiment, demonstrating the inhibitory effect of these drug concentrations on cell migration (*p* < 0.05, for both distance and area).

Finally, for the cultures treated with middle concentrations (15 and 20 µM), although they showed some progression of the proliferating fronts, the gap was not closed after 72 h, and there was a acellularized area of 0.725 × 10^6^ µm^2^ and 0.79 × 10^6^ µm^2^, respectively.

### 3.2. In Vivo Experiments

Once curcumin was proven to reduce cell proliferation in vitro, experiments were carried out to check if it was able to counteract the stimulatory effect of partial hepatectomy in vivo.

All of the animals inoculated with CC531 cells developed hepatic metastases disseminated throughout the whole liver. The effect of curcumin on tumor progression was quite striking (Figure 3 and Table 1), as the volume of the tumor masses was diminished to merely 20% of what was found in the animals receiving the vehicle (1.42 ± 1.34 cm^3^ vs. 7.99 ± 1.45 cm^3^; *p* < 0.0001). However, if we take a look at the volume of healthy liver parenchyma (Figure 3), it was significantly greater in the animals not receiving the drug (10.91 ± 1.01 cm^3^ vs. 8.8 ± 1.06 cm^3^; *p* < 0.01).

**Figure 4:**
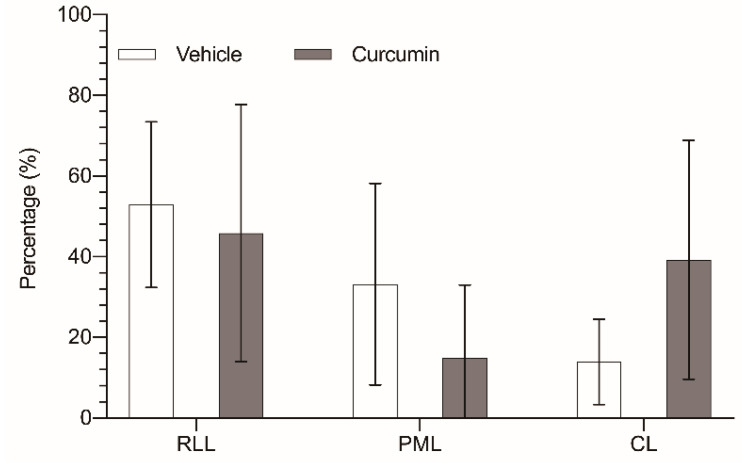
Percentage of tumor tissue in relation to the total liver mass of each lobe: right lateral lobe (RLL), paramedian lobe (PML), and caudate lobes (CL), removed from untreated animals (white bar) or animals treated with 200 mg/kg/day curcumin (grey bar). The results are expressed as mean and standard deviation (SD) from a single replicate (10 animals *per* group) and have been statistically analyzed by two-tailed t-test; no statistical significance is observed (*p* > 0.05).

Curcumin not only had diminished global tumor progression in the liver, but it has also changed the distribution of the tumor masses among the different liver lobes. In the control animals, the distribution of the tumor volume among the three remaining liver lobes did not correlate with the relative volume of those lobes. While the paramedian lobe (PML) accounts for 47.5% of the liver parenchyma, the tumor located in this lobe totaled only 33.2% of the tumor burden. On the other hand, right lateral lobe (RLL) had the highest tumor burden (52.9% of tumor tissue), while its parenchyma represented only 34.4% of the organ. Finally, the caudate lobes (CL) allocated 13.9% of the tumor volume in a 18% of liver parenchyma (Figure 4).

In the treated group, those differences were sharply noted, with a notable reduction in the tumor allocated in the PML (17.3% vs. 33.2%), while the tumor burden in the CL was more than doubled (36.8% vs. 13.9%); only the tumor burden of the RLL remained quite similar (45.9% vs. 52.9%). We also observed a slight variation of the distribution of the liver parenchyma among the three lobes (RLL 41.9%, PML 42.1%, and CL 16%), but these changes were not statistically significant, and in fact went in the opposite direction of the variation in the tumor distribution.

**Figure 5:**
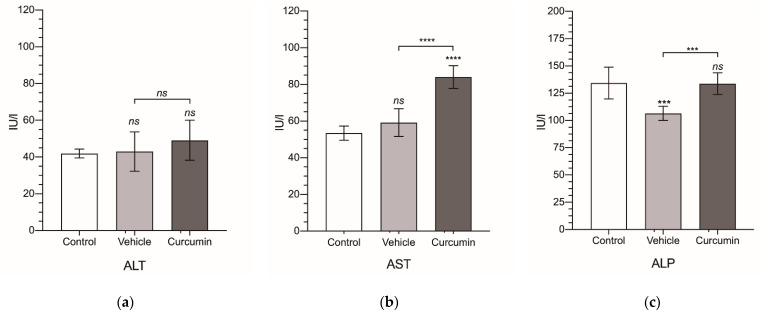

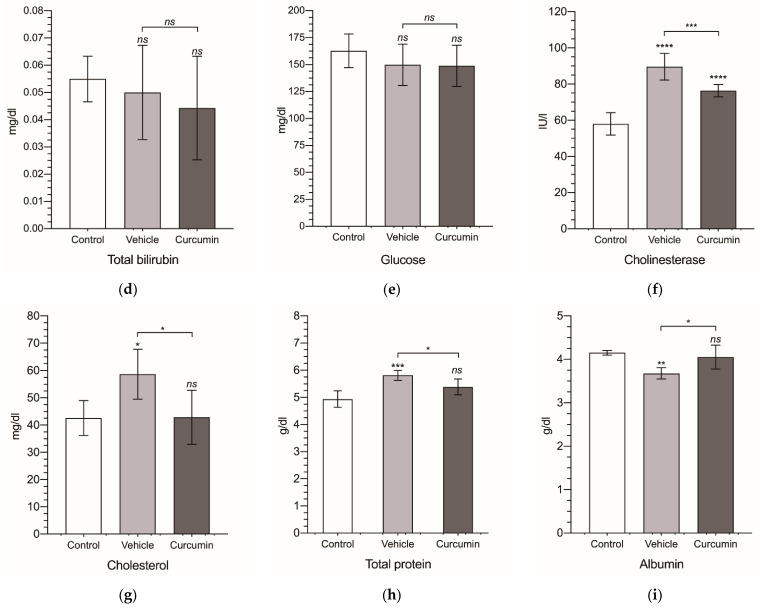
Biochemical serum levels. Alanine transaminase (ALT) (**a**), aspartate transaminase (AST) (**b**), alkaline phosphatase (ALP) (**c**), total bilirubin (TBil) (**d**), glucose (**e**), cholinesterase (**f**), cholesterol (**g**), total protein (TP) (**h**), and albumin (**i**). Levels are expressed in international units of activity per liter (IU/L) for ALT, AST, ALP, and cholinesterase; in milligrams per deciliter (mg/dL) for TBil, glucose, and cholesterol; and in grams per deciliter (g/dL) for TP and albumin. Results are expressed as mean and standard deviation (SD) of a single replicate (10 animals in the vehicle- and curcumin-treated group and 6 animals for the control group). The statistical analysis was carried out by a one-way ANOVA test, followed by Newman−Keuls multiple comparison test for between-group comparisons to the control group, or between the vehicle group and the curcumin group. Asterisks indicate statistical significance: *: *p* < 0.05; **: *p* < 0.01; ***: *p* < 0.001; **** *p* < 0.0001; ns: *p* > 0.05. Control group represents non-tumor-bearing and non-treated animals.

Finally, from the serum collected from all of the animals, we obtained a biochemical profile for each experimental group (Figure 5 and Table 2). In tumor-bearing animals receiving sesame oil, ALP and albumin were significantly decreased (Figure 5c,i), while the cholinesterase, cholesterol, and total protein were significantly increased (Figure 5f–h). These changes were completely reverted by curcumin, except for cholinesterase, where the significant reduction observed (*p* < 0.001) normal values were not reached. However, curcumin treatment was followed by a significant increase in AST (Figure 5b), not observed in the non-treated animals.

## 4. Discussion

As previously mentioned, curcumin accounts for a wide variety of biological activities, ranging from protection against bacterial infections or downregulating inflammatory responses to reducing tumor growth. Not surprisingly, we found curcumin to inhibit in vitro CC531 proliferation ant to significantly slow down tumor growth in vivo.

The underlying mechanism to curcumin’s antitumor effect is not yet clear. Exploring the antiproliferative effect of curcumin in vitro, Ramachandra [27] proved that tumor cells are more sensitive to curcumin than human mammary epithelial cells. He proposed that this differential effect could be explained by the different telomerase activity detected in both cell lines (6.9 times higher in tumor cells). In fact, the telomerase activity is overexpressed in approximately 85% of human cancers, and is very low in somatic cells [28]. However, Liu et al., finding that two different breast cancer cell lines (BT-483 and MDA-MB-231) required different concentrations of curcumin to achieve similar levels of cell proliferation [29], proposed that curcumin’s activity depended on down-regulating the NF-kB inducing genes, and different cell lines showed different dependences on this pathway. A third proposal was made by Chen et al., who found curcumin reduced pancreatic cancer cell migration. They reported an up-regulation of E-cadherin related to the suppression of invasiveness [30]. Mosieniak et al. demonstrated that this antitumor effect of curcumin was due to the fact that it leads to the senescence of colorectal cancer cells, and reported a functional connection within the senescence mechanism and autophagy activity in cells treated with 10 µM curcumin [7]. They also reported that cellular senescence is lower in p53−/− cells, which is correlated with a lower level of p21, a senescence marker in tumor cells.

The mechanism is not entirely clear, as several molecular pathways have been established, and neither the ideal dose or concentration of curcumin have been found. To assess the antitumor activity of curcumin, different approaches have been found in the literature. While many have focused on the proliferation rate of in vitro cultures, others have studied cell migration using so-called wound healing tests, and very scarcely has the effect of curcumin using in vivo animal models been analyzed.

In our in vitro experiments, the figures measured after 72h of curcumin exposure (25 or 30 µM) decreased to 40% of the control cultures. CC531 cells showed a higher sensitivity to curcumin than most of those studied by Ismail et al., which included normal human colon (CCD-18co) and hepatic (WRL-68) cell lines, and primary (SW480) and metastatic (SW620) human colon cancer cell lines. In these cells, curcumin only reduced the cell counts to 60 to 70%. Only in cultures of SW620 metastatic cells were the effects of the drug slightly similar to our results, decreasing the cell count to 45% [31].

Sanaei and Kavoosi also found that curcumin (5, 10, 25, and 50 µM) reduced up to 50% of the cell viability after 72 h of exposure, and also somehow induced apoptosis through epigenetic modification [32]. Lee and colleagues reported the benefit of curcumin treatment on medulloblastoma cells [33]. They reported, in a similar pattern to our results, that curcumin induced cell death in a time- and dose-dependent manner when treated with 10, 20, or 40 µM. Furthermore, they showed that curcumin increased the number of medulloblastoma cells arrested in the G2/M phase of the cell cycle 7 h after the start of treatment. In this sense, treating with 10 µM resulted in a higher percentage of cells arrested in the G2/M phase, which is well known to lead to increased cell apoptosis [34,35]. Regarding cell mobility, it is widely accepted that curcumin reduces cell migration. Chen et al. [30] found that the area occupied by CL1-5 human lung adenocarcinoma cells after 12 h of culture was reduced by 30, 50, and 75% when curcumin doses of 5, 10, and 20 µM, respectively, were added. These results are in accordance with those obtained by Yang, working on NCI-H446 and NCI-1688 human small cell lung cancer [36]. Other studies have reported similar findings for Patu8988 and Panc-1 pancreatic cancer cells [37,38]. Both groups, assessing invasion at 20 h, reported their greatest effect using 15 µM and 20 µM, which reduced the occupied area by 50% and 80%, compared to non-treated, respectively. Although we also found an inhibition of cell migration, when assessed at 20 h, the area occupied by migrating cells was not affected by curcumin, independently of the dose used; we had to wait another 10 h to find any significant modification in cell mobility. After 30 h, the area of the wound occupied by the migrating cells was reduced to 36.2% or 31.9% depending on the dose used (15 µM or 20 µM, respectively). However, our inhibition was greater than the one reported by Su and Zhang, as they did not continue their observations beyond 20 h, nor could any conclusion be reached about the sensitivity of our cells to curcumin.

Some in vivo experiments involving curcumin and cancer have been published. Odot showed that although curcumin (25 mg/kg/day, ip) does not reduce the number of animals developing melanoma metastases, a 40% reduction in tumor size was observed [39]. Byun reported similar results in a colitis-relate mouse colon carcinogenesis model, with a 40% reduction in the number of tumor foci, which were also smaller than those not treated with curcumin [40]. Lee et al. completed their in vitro studies with in vivo studies. In two animal models, they obtained similar results to Odot and Byun, showing that daily treatment with 1 g/kg curcumin suppressed tumor growth by 40% compared with the control group treated with the vehicle (corn oil) [33]. Kunnumakkara et al. also reported the beneficial effects of curcumin (1 g/kg, once daily orally) on colorectal cancer treatment. They showed that curcumin significantly enhanced the efficacy of fractionated γ-radiation therapy by suppressing NF-κB activation and NF-κB-regulated gene products, leading to the inhibition of proliferation and angiogenesis, implying a prolonged time to tumor relapse [41].

We achieved a greater reduction in tumor growth (80%), but the dose of curcumin administered was also greater (200 mg/kg/day). They have used an intraperitoneal administration, while we gave the drug orally. When the oral route is used, a lower bioavailability of curcumin is reached because of its low water solubility [42,43]. Thus, as neither of us assessed the plasma levels of the drug, a conclusion on dose-related effects cannot be obtained.

Another noteworthy fact is that these authors also reported an increase in mean survival of 45.7%. However, in our case, no animal deaths were recorded during the experiment.

Another notable finding in our results is the change in the growth pattern of metastases in those animals treated with curcumin compared with those that received the vehicle. In this matter, to the best of our knowledge, we cannot attribute this fact to any known factor, as we did not find similar results reported in the literature. It is true that in clinical practice the pattern of tumor recurrence is a determining factor for predicting the overall survival or for deciding the therapeutic strategy, either local or systemic therapy [44,45].

However, in the clinical setting, recurrence pattern refers to the presence of extrahepatic or intrahepatic disease; among the latter, a subclassification can be made between solitary or few nodules, or multinodular [46]. In our experiment, regardless of the experimental group, all animals developed intrahepatic multinodular implants, so, in our belief, the change in metastatic distribution pattern as a consequence of curcumin treatment may not have had a major transcendence.

These previously reported findings by Byun, Lee, Odot, and Kunnumakkara et al. make it clear that curcumin has a remarkable antitumor effect. However, in our opinion, these studies employ animal models that, although useful to determine aspects such as the molecular pathways that are modified by the administration of curcumin, are far from clinical settings. These models exert some characteristics that could impact the correct translation to a clinical setting with humans suffering from colorectal cancer liver metastases and undergoing partial hepatectomy. For example, the study reported by Byun involved a model of colon carcinogenesis induced by the administration of azoxymethane (AOM). This compound, as well as being well-known to induce colon carcinogenesis, may also have led to undesirable side effects, ranging from animal discomfort, with loss of appetite and weight loss, or minor toxicological reaction (hepatotoxic) along with additional pathological changes, including small intestinal tumors (especially in the duodenum), cholangiomas, and hepatocellular carcinomas, and even renal tumors [47,48]. These side effects, especially those related to the liver toxicity, infer in the hepatic regeneration process [49], so the results obtained by Byun et al. cannot be directly transferred to the clinical setting in humans.

On the other hand, the experimental model used by Odot and Kunnumakkara in their studies consisted of tumor xenografts in nude animals (female SCID mice and male athymic nu/nu mice, respectively) that lacked a fully competent immune system (CIS). As a result of the high number of mutations that tumor cells acquire, they express tumor-specific antigens that cannot be identified as not their own, and thus activate the immune system, ultimately leading to the killing of the cancer cells. Curcumin has also been shown to impact the composition of the different constituents of the tumor’s immune microenvironment, such that the immune microenvironment favors tumor killing [50]. For example, in patients suffering from colorectal or lung cancer, curcumin promotes the transformation of regulatory T cells into T helper cells [51,52]. Therefore, these results emphasize the importance of CIS as a defense mechanism against cancer development and progression, and highlight the significance of using competent animals in preclinical trials of new cancer therapies.

What should be highlighted from our experiment is that we were able to slow down tumor growth in the liver without severely impairing liver growth following partial resection of the organ. Hepatic resection, probably because of all the growth factors it generates, stimulates the growth of any metastases remaining in the liver [53,54], and this undesired effect cannot be reverted by chemotherapy as it hinders liver recovery [55,56,57]. Administering curcumin prior to a partial hepatectomy could be an interesting approach when resecting liver metastases, as it could avoid the stimulus over dormant cancer cells or micrometastases remaining in the liver. In addition, when performing selective portal vein ligation to increase liver mass prior resection of metastases, curcumin could help to inhibit tumor growth during liver regeneration. Although our biochemical analyses show that curcumin has returned the alterations induced by the tumor and the hepatectomy to normal values, we cannot ignore the slight elevation of AST and the delay in liver mass recovery found in the curcumin treated animals [58]. In some cases, these marked increases in AST activity, coupled with a minimal or slight change in ALT activity, as occurred in our study, may indicate that the AST released into the bloodstream arises from non-hepatic sources, such as the skeletal muscle, heart, or kidneys [59]. Another feasible explanation, stated by Vuppalanchi y Chalasani [60], is that this is a result of the presence of the macro-AST enzyme. In this setting, AST forms complexes with other proteins, mainly immunoglobulin G (IgG), resulting in a chronically elevated AST level, which has no clinical consequences. However, to prove this, other tests should be done that we did not have the ability to do.

If we compare our results with those previously published on the effect of well-known chemotherapeutic compounds on CC531 cultures [61,62,63], we can see that curcumin exerts, at least, a similar tumor inhibition. We obtained a 50% reduction in cell viability with 20 µM curcumin after 72 h. Van Putte [61] reached a 50% reduction after 96 h using cisplatin (10.3 mM), gemcitabine (7.6 mM), or melphalan (8 mM). Herol, using ciprofloxacin (200 and 500 μg/mL), inhibited DNA synthesis in CC531 cells, and also led to apoptosis in a time-dependent manner, reaching 73 and 68% of apoptotic cells after treatment [62]. Meanwhile, Ocker, analyzing the effect of the synthetic retinoid adapalene (ADA) and 9-cis-retinoic acid (CRA) on CC531 cell lines, stated that both retinoic derivatives suppressed DNA synthesis and induced apoptosis. The strongest anti-proliferative and pro-apoptotic effect was found with 10^−4^ M after 72 h of exposure, reaching a percentage of apoptotic cells of 75% and 41%, respectively [63].

A similar parallelism can be found with in vivo assays using CC531 and chemotherapy. Maneikyte reported that FOLFOX did not significantly reduce the size of CC531-induced tumor implants after 14 days of development and two doses of FOLFOX compared to the control [64]. The failure of FOLFOX in this tumor model may be due to the tumor induction mechanism and the timing of the analysis after tumor induction. Maneikyte used a subcapsular injection model that induced the development of a single, well-defined, and poorly vascularized metastasis [65], which may condition the successful delivery of the drug to the whole tumor mass. On the other hand, we analyzed tumor response after 28 days, which means that our results and those of Maneikyte are not fully scalable. Finally, our results match those reported by Heskamp and colleagues [66]. They induced tumor implants by injecting CC531 cells, which were then treated with four cycles of 5-FU at different doses (15, 30, and 60 mg/kg). The two highest doses approximately halved or completely reduced the number of tumor foci. Tumor size was also reduced from 4 g (vehicle) to 0.5 and 0 g (30 and 60 mg/kg, respectively).

It seems that after all the reported data, it can be accepted that curcumin has a relevant antitumor effect. The next step to make it a relevant treatment for those patients suffering from colorectal cancer liver metastases would be to check its use in combination with drugs currently used in patients, such as 5-FU, irinotecan, oxaliplatin, or their combinations, or with therapies based on monoclonal antibodies, such as bevacizumab [67]. This aspect could be especially beneficial in cases of tumors resistant to these therapies [68]. In this sense, focusing on the results obtained in tumors of the gastrointestinal tract, there are several papers where the potential synergistic effect of curcumin has been proposed. Shakibaei et al. reported that as low as 5 µM curcumin increased the chemosensitivity of mismatch repair (MMR)-resistant human colorectal cancer cells to 5-FU. They also found that curcumin reverted the multidrug resistance observed in MMR-resistant human colorectal cancer cells [6]. In vivo studies also support the idea of using curcumin together with currently used drugs. For example, Zhou et al. [5] demonstrated that curcumin/5-FU exerts a potent inhibitory effect on BGC-823 xenograft tumors on nude mice, by reducing its volume by 25%. Yang et al., working on nude mice bearing MKN45 tumor xenografts in vivo, demonstrated that the combination of curcumin with 5-FU resulted in an 80% smaller tumor size than with 5-FU treatment alone (258 ± 38 vs. 1235 ± 105 mg, respectively) [69]. On the other hand, this synergistic effect of curcumin goes beyond the gastrointestinal tract. Zhan et al. demonstrated that curcumin enhances the benefits of platinum-containing chemotherapeutic agents for NSCLC treatment in athymic nu/nu (BALB/c) mice; the inhibitory effect on tumor volume of cisplatin in combination with curcumin was twice that of cisplatin alone [70]. Furthermore, the combination of curcumin with drugs used for purposes other than tumor treatment, such as metformin, achieved an increase in the antiangiogenic effect, decreasing tumor size in breast cancer in mice [71].

## 5. Conclusions

Curcumin has shown an anti-tumor effect against liver implants from colorectal cancer, both in vitro and in vivo, in this experimental model.

The knowledge accumulated so far about the effects of curcumin on tumor development and progression seems relevant enough to foster some kind of clinical trial, either in liver metastasectomies or in selective portal branch ligation prior to hepatic tumor resection.

## Figures and Tables

**Table 1:** Total liver parenchymal and tumor tissue volume values, as well as detailed values for the right lateral lobe (RLL), paramedian lobe (PML), and caudate lobes (CL) (cm^3^) are expressed as mean and standard deviation. The figure in brackets expresses the percentage of each lobe relative to the total mass of the liver or tumor, depending on the tissue type.

Group	Sesame Oil-Treated Animals				Curcumin-Treated Animals			
	Total	RLL	PML	CL	Total	RLL	PML	CL
Liver parenchyma	10.9 ± 1.01	3.76 ± 1.5 (34.5%)	5.18 ± 1.4 (47.5%)	1.97 ± 0.7 (18%)	8.80 ± 1.06	3.69 ± 0.53 (42%)	3.7 ± 0.89 (42%)	1.41 ± 0.356 (16%)
Tumor tissue	7.99 ± 1.45	4.30 ± 2.27 (53.8%)	2.59 ± 2.06 (32.4%)	1.1 ± 0.86 (13.8%)	1.42 ± 1.34	0.67 ± 0.62 (47.2%)	0.11 ± 0.14 (7.7%)	0.64 ± 1.09 (45.1%)

**Table 2:** Biochemical serum values for alanine transaminase (ALT), aspartate transaminase (AST), alkaline phosphatase (ALP), total bilirubin (TBil), glucose, cholinesterase, cholesterol, total protein (TP), and albumin. Levels are expressed in international units of activity per liter (IU/L) for ALT, AST, ALP, and cholinesterase; in milligrams per deciliter (mg/dL) for TBil, glucose, and cholesterol; and in grams per deciliter (g/dL) for TP and albumin.

Group	ALT	AST	ALP	TBil	Glucose	Cholinesterase	Cholesterol	TP	Albumin
Control	41.9 ± 2.43	53.5 ± 3.83	134 ± 14.5	0.055 ± 0.008	163 ± 15.5	58.0 ± 6.24	42.6 ± 6.43	4.94 ± 0.305	4.15 ± 0.06
Vehicle	43.0 ± 10.7	59.2 ± 7.52	107 ± 6.47	0.05 ± 0.017	150 ± 19.2	89.6 ± 7.35	58.7 ± 9.14	5.82 ± 0.183	3.68 ± 0.13
Curcumin	49.1 ± 10.9	84.0 ± 6.13	134 ± 9.95	0.044 ± 0.019	149 ± 19.1	76.4 ± 3.38	42.9 ± 9.94	5.39 ± 0.291	4.05 ± 0.27

## Data Availability

The data that support the findings of this study are available from the corresponding author, (B.H.d.l.P), upon reasonable request.

## Supplementary Materials

The following are available online at https://www.mdpi.com/article/10.3390/biomedicines9091183/s1. Figure S1: Graphical representation of timeline of the tumor induction and treatment. Figure S2: Cell proliferation of CC531 rat colorectal cancer cells as a function of time. Figure S3: Representative phase-contrast pictures of both control and treated-cells with the various concentrations of curcumin (15, 20, 25, and 30 µM) at 0 h (immediately after removing the insert) and 20, 30, 44, 54, and 72 h after the scratch are shown. Table S1: Fluorescence absolute values.
