# Supplementary material for: Curcumin Reduces Colorectal Cancer Cell Proliferation and Migration and Slows In Vivo Growth of Liver Metastases in Rats
Source: Biomedicines. 2021 Sep 8;9(9):1183. doi: 10.3390/biomedicines9091183 (PMC8467247; doi:10.3390/biomedicines9091183)
Supplement: Supplementary file 1 [file biomedicines-09-01183-s001.zip › biomedicines-1264304-supplementary.pdf]

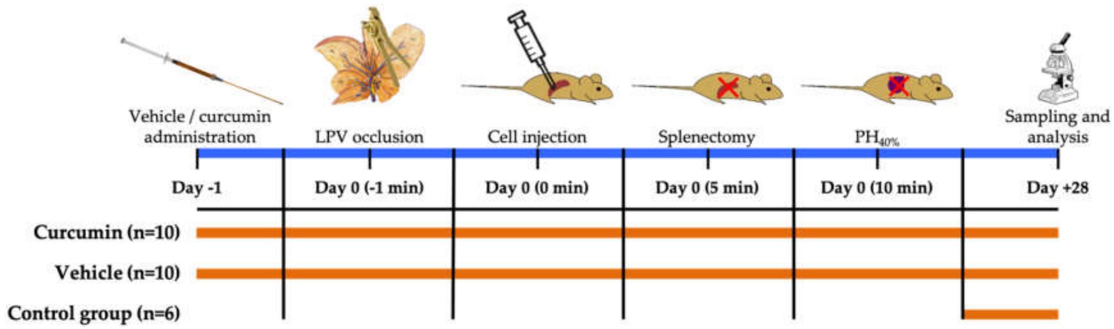

Figure S1. Graphical representation of timeline of the tumor induction and treatment. Note that the administration of curcumin or vehicle began on day −1 and ended on day 28. The control group did not undergo any experimental process, only samples were taken to determine the baseline values of the parameters analyzed. LPV—left portal vein; PH—partial hepatectomy.

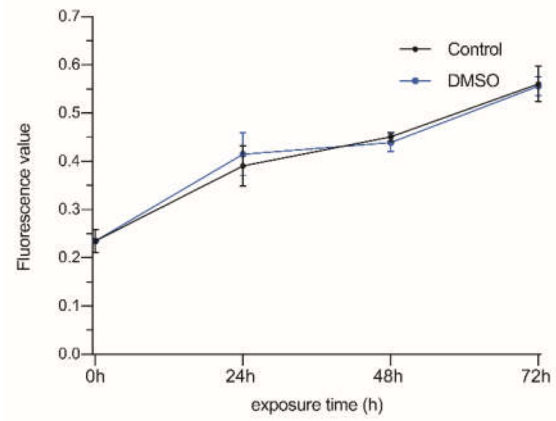

(**a**)

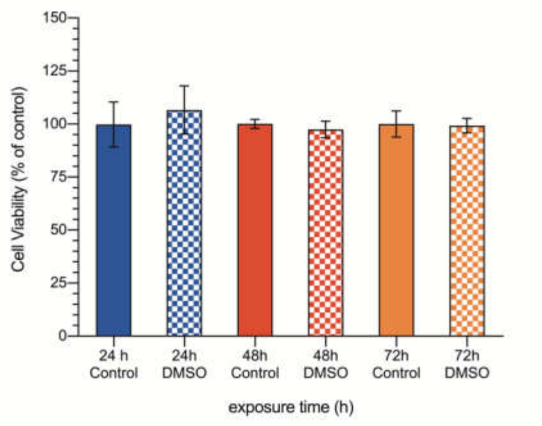

(**b**)

Figure S2. Cell proliferation of CC531 rat colorectal cancer cells as a function of time. Absolute fluorescence values (A) and percentage of control (B) of the control and cultures supplemented with DMSO (as a vehicle for curcumin treatment at 24, 48, and 72 h). The dotted line of (A) shows fluorescence values prior to curcumin exposure.

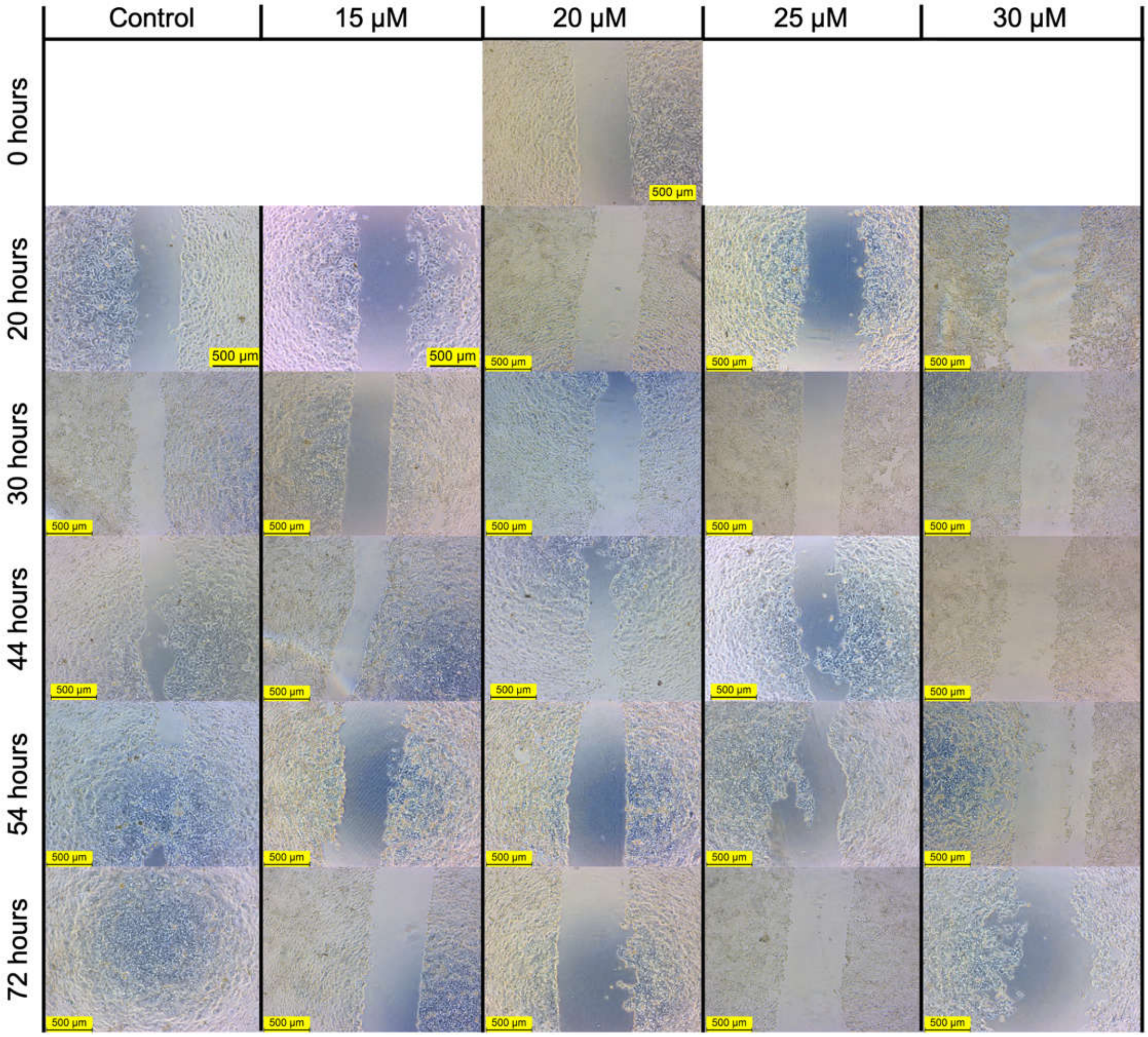

Figure S3. Representative phase-contrast pictures of both control and treated-cells with the various concentrations of curcumin (15, 20, 25, and 30 μM) at 0 h (immediately after removing the insert) and 20, 30, 44, 54, and 72 h after the scratch are shown. Scale bar: 500 μm

Table S1. Fluorescence absolute values. Arbitrary units of fluorescence (AU) measured in control cultures and those exposed to 15, 20, 25, and 30 μM of curcumin at 0, 24, 48, and 72 h. Values are presented as mean and standard deviation.

[Curcumin]	0 h (AU)	24 h (AU)	48 h (AU)	72 h (AU)
**Control**		0.403 ± 0.122	0.443 ± 0.059	0.558 ± 0.087
**15 μM**		0.426 ± 0.084	0.319 ± 0.040	0.304 ± 0.094
**20 μM**	0.235 ± 0.095	0.394 ± 0.054	0.302 ± 0.061	0.288 ± 0.073
**25 μM**		0.389 ± 0.102	0.274 ± 0.061	0.235 ± 0.081
**30 μM**		0.306 ± 0.108	0.243 ± 0.028	0.222 ± 0.088
